# Modulation of *Staphylococcus aureus* spreading by water

**DOI:** 10.1038/srep25233

**Published:** 2016-04-29

**Authors:** Mei-Hui Lin, Wan-Ju Ke, Chao-Chin Liu, Meng-Wei Yang

**Affiliations:** 1 Department of Medical Biotechnology and Laboratory Science, College of Medicine, Chang-Gung University, Taoyuan, 333, Taiwan; 2 Department of Laboratory Medicine, Chang-Gung Memorial Hospital, No. 5, Fusing St., Guishan, Taoyuan, 333, Taiwan; 3 Research Center for Bacterial Pathogenesis, Chang-Gung University, Taoyuan, 333, Taiwan; 4 Department of Microbiology and Immunology, Chang-Gung University, Taoyuan, 333, Taiwan

## Abstract

*Staphylococcus aureus* is known to spread rapidly and form giant colonies on the surface of soft agar and animal tissues by a process called colony spreading. So far, the mechanisms underlying spreading remain poorly understood. This study investigated the spreading phenomenon by culturing *S. aureus* and its mutant derivatives on Tryptic Soy Agarose (TSA) medium. We found that *S. aureus* extracts water from the medium and floats on water at 2.5 h after inoculation, which could be observed using phase contrast microscopy. The floating of the bacteria on water could be verified by confocal microscopy using an *S. aureus* strain that constitutively expresses green fluorescence protein. This study also found that as the density of bacterial colony increases, a quorum sensing response is triggered, resulting in the synthesis of the biosurfactants, phenolic-soluble modulins (PSMs), which weakens water surface tension, causing water to flood the medium surface to allow the bacteria to spread rapidly. This study reveals a mechanism that explains how an organism lacking a flagellar motor is capable of spreading rapidly on a medium surface, which is important to the understanding of how *S. aureus* spreads in human tissues to cause infections.

*Staphylococcus aureus* is known to form giant colonies when cultured on soft TSA medium in a process called colony spreading, which was first reported by Kaito and Sekimizu^1^. When cultured on TSA medium that contains 0.24% (TSA-0.24) agar, *S. aureus* is capable of expanding its colony at a speed of approximately 100 μm per minute, although the bacteria are unable to spread when the agar concentration is increased to 1.5%^1^. The ability to move at this speed is amazing, since the organism lacks flagellum to allow itself to move actively on the agar surface. They also showed that mutations in the *dltABCD* operon and *tagO* gene, which are involved in adding D-Ala to teichoic acid^2^ and the synthesis of teichoic acid^3^, respectively, disable the spreading ability of *S. aureus*, indicating the importance of teichoic acid in spreading^1^. Additionally, *S. aureus* spreading requires the synthesis of phenol-soluble modulins (PSMs), which are cytolytic toxins that have surfactant properties^4^,^5^,^6^,^7^; among the 8 PSMs produced by *S. aureus*, PSMα3 is particularly important for spreading^6^. Earlier studies also found that mutations in the genes encoding proteins in the Agr quorum-sensing system prevent spreading^7^,^8^. However, the lack of ability to spread could be reverted if PSMs are added to the culture medium^6^. Since the transcription of the *psm* genes is activated by the quorum sensing activator, AgrA^9^, the results indicated that the lack of PSMs expression contributes to the inability of the quorum-sensing mutants to spread. Additionally, cell wall-associated factors such as fibronectin binding proteins and clumping factors, which promote biofilm formation, antagonize colony spreading of *S. aureus*^10^. Spreading was also found to be inhibited by the secretion of δ-hemolysin^11^ but stimulated by bovine serum albumin and high density lipoprotein in the serum^12^.

Swarming bacteria are found to extract water from agar medium^13^,^14^. Although a study suggested that *Escherichia coli* uses lipopolysaccharides (LPS) as osmolytes to extract water from agar^15^, the mechanisms involved in water extraction are not completely understood. However, the combination of water extraction from agar medium and the use of biosurfactants to facilitate swarming is well documented in *B. subtilis*^16^. This study shows that although *S. aureus* cannot move actively, the bacterium uses a mechanism similar to that of *B. subtilis* to extract water from agar medium and expresses biosurfactants to weaken the water surface tension to facilitate colony spreading.

## Results

### Spreading of *S. aureus* HG001

**Figure 1:**
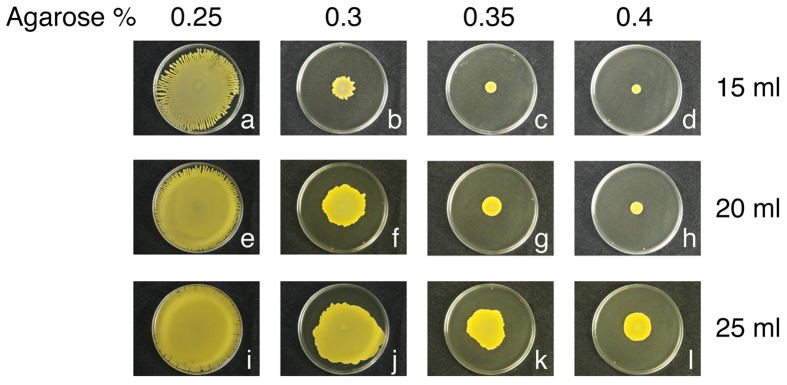
Spreading of *S. aureus* HG001 on TSA medium. An overnight culture of *S. aureus* HG001 cultured in 3 ml TSB was washed with the same medium volume of PBS before inoculation. TSA plates (15 ml, 20 ml, and 25 ml) that contained 0.25, 0.3, 0.35, and 0.4% agarose were inoculated with 2 μl of the bacteria. The plates were incubated at 37 °C for 24 h.

*S. aureus* is known to spread on the surface of TSA medium containing 0.24% agar (TSA-0.24)^1^. Since *S. aureus* does not have a flagellum or any other surface apparatus that would allow the bacterium to power its movement, we posit that *S. aureus* uses water and moves passively across the medium surface during spreading. *S. aureus* spreading is commonly studied on TSA-0.24 agar^1^,^6^,^7^. A medium with agar at this concentration contains large amounts of water, therefore, studying water extraction by *S. aureus* on a plate surface that is already saturated with water can be difficult. Additionally, types of agar used to prepare TSA were found to influence not only the ability but also the speed of *S. aureus* HG001 spreading. To determine if a plate with a relatively dry surface could be utilized for assaying water extraction, we prepared TSA plates with agarose (SeaKem) to determine how the concentration of agarose and volume of the medium affected the spreading of *S. aureus* HG001. We found that when the strain was cultured on a 15-ml TSA plate that contained 0.25% agarose (TSA-0.25) in a 9-cm petri plate, *S. aureus* HG001 spread and its colony covered almost the entire plate with numerous tendrils developing at the colony’s edge after 24 h (Fig. 1a). When the agarose concentration was increased to 0.3% (TSA-0.3), the ability of the bacterial spreading was reduced; the size of the colony on this plate was approximately 1.5 cm in diameter (Fig. 1b). When the percentage of agarose was increased further to 0.35% or 0.4% (TSA-0.35 or TSA-0.4, respectively), the strain did not spread (Fig. 1c,d). We also found that increasing the volume of TSA medium in the plate could compensate for the inability of *S. aureus* to spread on plates containing a high percentage of agarose. When the volume of the medium was increased from 15 ml to 20 ml, the bacteria formed a colony that was larger and thicker than that formed on a 15-ml TSA-0.25 plate (Fig. 1a,e), and the bacteria were able to spread on TSA-0.3 and TSA-0.35 plates (Fig. 1f,g). We also found that the bacteria were unable to spread on a TSA-0.4 plate with 20 ml of TSA medium (Fig. 1h). When the volume of TSA was increased to 25 ml, the bacteria spread on TSA plates that contained 0.25–0.4% agarose (Fig. 1i–l). Therefore, in this study, we used 25-ml TSA-0.4 plates for studies on water extraction by *S. aureus*.

### Phase contrast microscopic observation of *S. aureus* HG001 colonies

We observed *S. aureus* HG001 colonies formed on 25-ml TSA-0.4 plates under a phase contrast microscope at a magnification of 400x. We found that 20 min after inoculation, the colony was flat and bacteria were distributed sparsely within the colony. Many bacteria were found oscillating, typical of Brownian movement (Supplementary Video S1), suggesting that a small amount of water was present in the colony causing the movement. At 2.5 h after inoculation, the morphology of the colony changed significantly. Under the microscope, bacteria at the center of the colony were found in different depths. In the colony, a few bacteria at the bottom appeared to be stationary; those on the top were moving (Supplementary Video S2), showing the presence of water in the colony. Most importantly, although the bacteria were moving in water, they moved passively as the bacteria were all moving in a general direction at the same speed (Supplementary Video S2), suggesting that bacteria in the colony were floating and drifting in flowing water. After the bacteria had been cultured for 4 h, we found that a colony was densely packed by bacteria but were still moving in the same general direction (Supplementary Video S3).

### Generating mutants that are defective in quorum sensing and spreading

**Figure 2:**
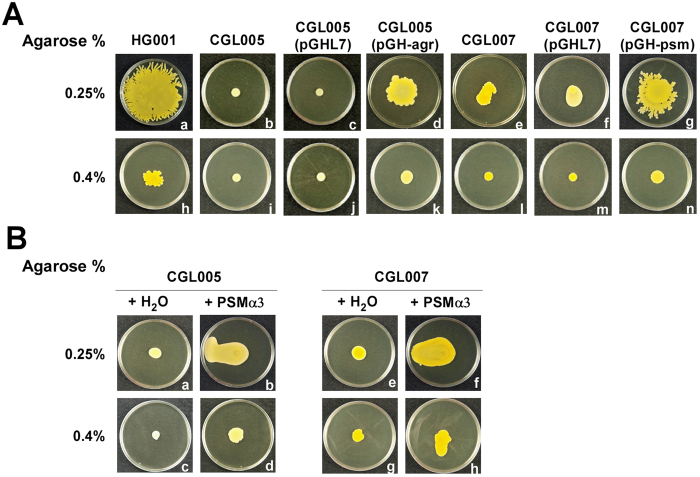
Influence of Agr system and PSMα on colony spreading. (**A**) *S. aureus* HG001, its *agrD*-deficient mutant, CGL005, *psmα*-deletion mutant, CGL007, CGL005(pGH-agr), and CGL007(pGH-psm) were cultured on 25 ml TSA-0.25 (a–g) and 25 ml TSA-0.4 (h–n) plates at 37 °C for 24 h. The mutants containing empty vector, i.e. CGL005(pGHL7) and CGL007(pGHL7), were used as controls. (**B**) Water (a,c,e,g) or 1 mM PSMα3 (b,d,f,h) was applied to the center of 25 ml TSA-0.25 (a,b,e,f) and 25 ml TSA-0.4 (c,d,g,h) plates. After drying for 30 min, 2 μl CGL005 (a–d) or CGL007 (e–h) was applied to the plates. Bacteria were cultured at 37 °C for 24 h.

We have mutagenized *S. aureus* HG001 using the *bursa aurealis* transposon^17^. Among the 2000 mutants screened, four mutants did not expand their colony on 25 ml TSA-0.25 plates after 24 h of culturing. Sequencing of these mutants revealed that mutants CGL005 and CGL006 had a transposon insertion in *agrD* (Supplementary Fig. S1) and *agrC*, respectively, which are genes involved in activating quorum sensing in *S. aureus*^18^. We found that CGL005 did not spread on TSA-0.25 and TSA-0.4 plates (Fig. 2Ab,i). After CGL005 was transformed with pGH-agr, which contains *agrD*, the spreading ability of CGL005 was restored (Fig. 2Ad,k). Furthermore, CGL005 expanded its colony on the plate that contained PSMα3, which was shown to be an important PSM for staphylococcal spreading^6^ (Fig. 2Ba–d). Since transcription of *psm* genes is activated by the Agr quorum-sensing system^9^,^19^, the addition of synthetic PSMα3 necessary for spreading showed that the lack of PSM synthesis by CGL005 was responsible for the mutant phenotype. This study also generated a mutant, CGL007, which has its *psmα* operon deleted, to demonstrate the importance of PSMα to spreading. The spreading ability of CGL007 on TSA-0.25 and TSA-0.4 plates was impaired (Fig. 2Ae,l), as compared with the spreading activity exhibited by the wild-type strain HG001 (Fig. 2Aa,h), due to the lack of PSMα synthesis. The spreading ability of CGL007 was restored when CGL007 was transformed with pGH-psm, which contains the whole *psmα* operon (Fig. 2Ag,n), but was not restored after the cells were transformed with an empty vector, pGHL7 (Fig. 2Af,m). Moreover, the spreading of CGL007 was promoted by adding PSMα3 onto TSA-0.25 and TSA-0.4 (Fig. 2Be–h). These results demonstrated that the Agr quorum-sensing system, which controls PSMs synthesis, is important to spreading by *S. aureus* HG001. The results are consistent with that reported previously^5^,^6^,^7^,^8^.

### Presence of water in colonies

**Figure 3:**
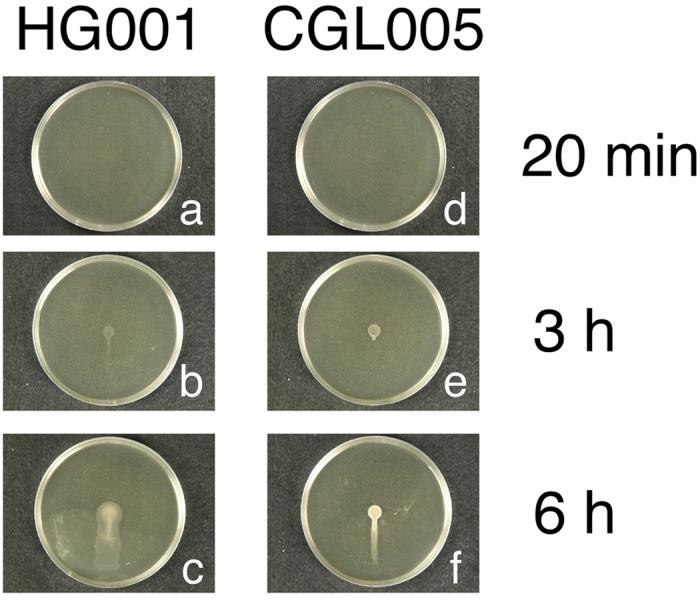
Water in *S. aureus* strains HG001 and CGL005. TSA-0.4 plates were inoculated with *S. aureus* HG001 (**a–c**) or CGL005 (**d–f**). The plates were laid at a 30° angle during culturing to allow water in the colonies to flow with gravity.

We cultured *S. aureus* strains HG001 and CGL005 on 25 ml TSA-0.4 plates and tilted the plates at a 30° angle during incubation. We found that at Hour 3, water started to flow and carry bacteria out of the HG001 colony (Fig. 3b); flowing of water was not observed for the CGL005 strain (Fig. 3e). This was not attributed to the lack of water in the mutant colony, since under a confocal laser-scanning microscope, the present of water in the colony was evident (data not shown). At Hour 6, a significant amount of water flowed out of the HG001 colony and accumulated at the bottom of the plate (Fig. 3c). However, only relatively small amounts of bacteria flowed out of CGL005 colony with no water accumulating at the bottom of the plate (Fig. 3f). These results showed that without an activated quorum sensing system resulting in the inability to synthesize PSMs, water surface tension holds water and prevents water from flowing out of the CGL005 colony.

### Confocal laser-scanning microscopy of colonies formed by *S. aureus*

**Figure 4:**
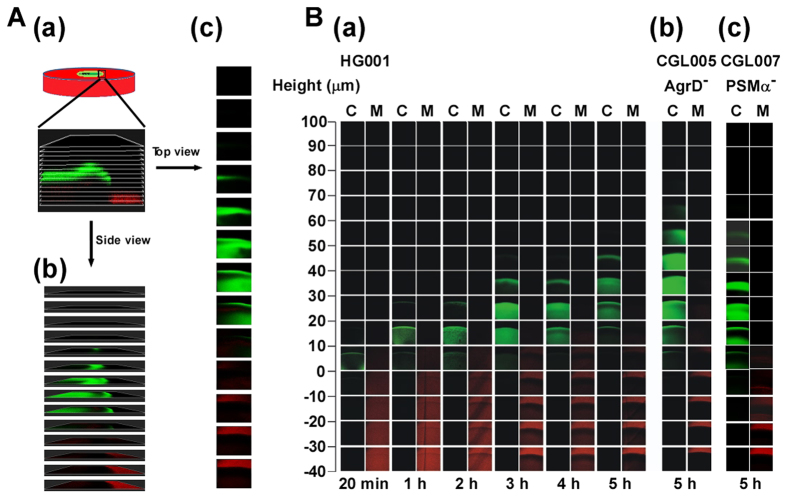
Height of colonies formed by *S. aureus* HG001, CGL005 and CGL007. (**A**) A diagram depicts the side view (b) and top view (c) images acquired from Z stacks of the edge of the colonies with planes separated by 10 μm (a) under a confocal laser-scanning microscope. Green fluorescence indicates the distribution of bacteria in the colony. The plate contained carboxylate-modified FluoSphere beads and the last plane with red fluorescence indicates the position of medium surface. (**B**) TSA-0.4 plates were inoculated with PBS-washed *S. aureus* HG001(pRPO-gfp) (a), CGL005(pRPO-gfp) (b) and CGL007(pRPO-gfp) (c). After incubating at 37 °C for the time indicated, the top view images of Z stacks of the colonies were acquired under a confocal laser-scanning microscope. C: colony, M: medium.

To measure the height of colonies, strains expressing GFP constitutively were inoculated on 25 ml TSA-0.4 plates and cultured at 37 °C. At each time point, the edge in the colony on the plate was observed (Fig. 4Aa). The Z stacks in which the planes were separated by 10 μm were acquired from the top to the bottom of the colony using an upright laser-scanning confocal microscope (Leica, TCS-SP2) (Fig. 4Aa). Green fluorescence in the micrographs indicated the distribution of bacteria in the spreading colony. The 0.5 μm carboxylate-modified FluoSphere beads (Ex580/Em605) (Invitrogen) were also incorporated into the medium to demarcate the plate surface. An illustration showed images of top and side views from the Z stacks (Fig. 4Ab,c), which were generated with Imaris software (Bitplane).

We cultured *S. aureus* HG001(pRPO-gfp) on 25 ml TSA-0.4 plates. Z stacks of the colonies were acquired under a confocal laser-scanning microscope (Fig. 4Ba). We found that 20 min after inoculation, the colony was thin and approximately 10 μm high (Fig. 4Ba). At Hours 1 and 2, growth of the bacteria was evident as the intensity of green fluorescence in the colony increased substantially. Meanwhile, the height of the colony increased to 30 μm (Fig. 4Ba). At Hours 3 and 4, the height of the colony increased to 40 μm (Fig. 4Ba); at Hour 5, the height of the colony increased to 50 μm (Fig. 4Ba). We found that at Hour 5 after inoculation, the colony formed by CGL005(pRPO-gfp) was 70 μm high with an intensity of green fluorescence substantially stronger than that of HG001(pRPO-gfp) (Fig. 4Bb). Macroscopic observation also showed that the HG001(pRPO-gfp) colony expanded but CGL005(pRPO-gfp) expanded very little during the five-hour period. The results indicated that the cell density and the height of colony formed by CGL005(pRPO-gfp) are higher than that formed by HG001(pRPO-gfp), which is due to a lack of PSMs synthesis to reduce the water surface tension of the colony and to restrict the colony expansion. We also examined the colonies formed by CGL007(pRPO-gfp), which has a defect in synthesis of PSMα. At Hour 5, the height of the colony was 60 μm (Fig. 4Bc), which was lower than the colony formed by CGL005(pRPO-gfp) but higher than HG001(pRPO-gfp).

### 3-D analysis of the *S. aureus* HG001 colonies

**Figure 5:**
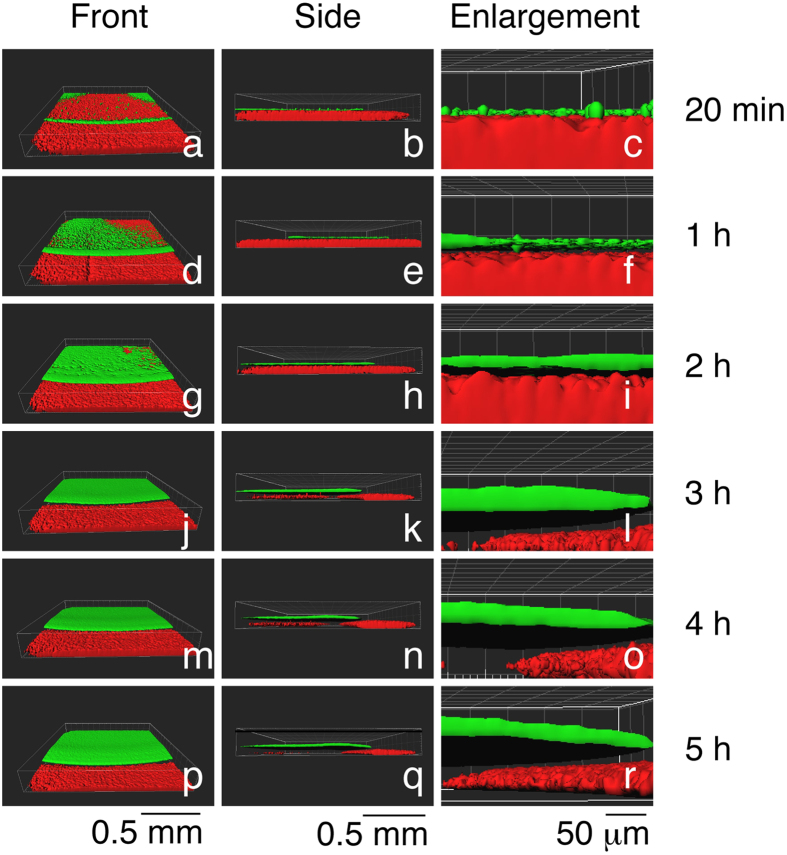
Floating of *S. aureus* HG001(pRPO-gfp) in colonies. 3-D images of *S. aureus* HG001(pRPO-gfp) colonies were generated from the Z stacks shown in Fig. 4B(a) using Imaris software. Parts in (b,e,h,k,n,q) were enlarged and shown respectively in (c,f,i,l,o,r). Green shows the distribution of bacteria; red shows the medium.

We generated 3-D images of *S. aureus* HG001 colonies using the confocal 2-D images shown in Fig. 4Ba and Imaris software (Bitplane). We found that 20 minutes after inoculation, bacteria were distributed sparsely inside the colony with a ring of accumulation at the colony edge (Fig. 5a). At Hour 1, bacterial growth was evident as more green fluorescence was found within the colony (Fig. 5d). At Hour 2, most of the areas in the colony were covered by bacteria (Fig. 5g). A thick layer of bacteria developed after Hour 3 (Fig. 5j,m,p). Images generated from the side of the colony revealed that bacteria were attached to the plate surface at 20 min after inoculation (Fig. 5b,c). At Hour 1, a space of several μm separated the green bacteria and the red medium (Fig. 5f), showing that bacteria were floating. During Hours 2–5, the green layer thickened and the distance between bacteria and the plate surface increased (Fig. 5g–r). These results showed that bacteria both grow and float in colonies.

### Quorum sensing and *S. aureus* spreading

**Figure 6:**
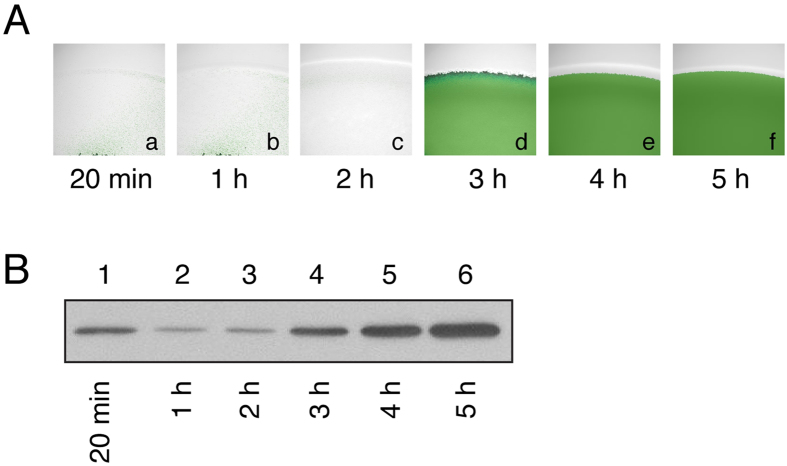
Occurrence of quorum sensing in spreading colony formed by *S. aureus* HG001. The log phase *S. aureus* HG001(pPSM-gfp) was applied to the surface of TSA-0.4 plates. Green fluorescence exhibited by the colony was observed under a confocal laser-scanning microscope (**A**). Cell lysates were prepared and GFP in the lysates were detected by immunoblotting with anti-GFP antibody.

Earlier studies demonstrated that mutants with mutations in *agr* genes do not spread on soft agar^6^,^7^,^8^, suggesting that quorum sensing is required for colony spreading of *S. aureus.* It is well known that expression of PSMs is activated by Agr quorum-sensing system^9^,^19^. However, it is currently unknown whether the quorum sensing response occurs in a spreading colony to modulate the spreading behavior. Therefore, a reporter plasmid pPSM-gfp, which contains the promoter of the *psm*α operon, which requires quorum-sensing activator (AgrA) for transcription, fused with a *gfp* reporter gene, was used in this study. We applied 2 μl log phase HG001(pPSM-gfp) cells (1 × 10^7^ CFU) to TSA-0.4 plates. We found that cells did not exhibit much green fluorescence before Hour 2 under a confocal laser-scanning microscope (Fig. 6Aa–c). However, strong green fluorescent signals were detected after Hour 3 (Fig. 6Ad–f). We also analyzed GFP expression by immunoblotting. To obtain enough proteins for the analysis, we had to inoculate 100 μl of the cells, which contained 5 × 10^8^ CFU, on one plate. The cells were harvested and homogenized. The lanes in a gel was loaded with 5 μg of the cell lysate. The results showed that 20 min after inoculation, the amount of GFP was relatively high (Fig. 6B, lane 1). This could be due to the long half-life of GFP and GFP being present in the cells prior to inoculation. However, the levels of GFP decreased at Hours 1 and 2 (Fig. 6B, lanes 2,3) but increased after Hour 3 (Fig. 6B, lanes 4–6). These results suggest that quorum sensing occurred before or at Hour 3, resulting in the activation of the *psm* promoters.

## Discussion

*S. aureus* is known to spread on a soft agar surface with an astonishing speed of approximately 100 μm per minute^1^, which parallels or exceeds the swarming speeds of many bacteria^20^. However, the mechanism of spreading by *S. aureus* differs from that of swarming because *S. aureus* lacks a flagellar motor to drive itself^1^. Therefore, the purpose of this study was to elucidate the underlying mechanism by which *S. aureus* spreads on a soft agar surface.

Although the work by Kaito and Sekimizu^1^ showed that *S. aureus* spreads only on TSA-0.24 plates, we found that *S. aureus* HG001 also spreads on TSA-0.4 plates (Fig. 1). We found that on this type of plate, *S. aureus* HG001 formed a colony filled by water (Supplementary Video S2) with a height of approximately 30–40 μm as measured by confocal microscopy (Fig. 4Ba). Additionally, water flowed out of the colony formed on a TSA-0.4 plate after the plate was tilted during incubation (Fig. 3a–c), verifying the presence of water in the colony. The results suggest that *S. aureus* HG001 is capable of extracting water from the medium. Earlier studies showed that the synthesis of PSMs is required for *S. aureus* to spread^5^,^6^,^7^. Therefore, we generated an *agrD* mutant, CGL005, which is unable to synthesize PSMs. We found that the colony formed by CGL005(pRPO-gfp) at Hour 5 was at least 40% or 20 μm higher than that formed by HG001(pRPO-gfp) (Fig. 4Ba,b). Due to a lack of colony expansion, the green fluorescence in the CGL005(pRPO-gfp) colony was more intense with a higher bacterial density than the green fluorescence in HG001(pRPO-gfp) (Fig. 4Ba,b). The defect in spreading of CGL005 can be complemented with a plasmid that expressed AgrD (Fig. 2A), or accomplished by adding synthetic PSMα3 to the plate (Fig. 2B). Furthermore, less water flowed out of the CGL005 colony than that from a HG001 colony after 3 and 6 h of incubation in tilted plates (Fig. 3d–f), showing that PSMs are required to weaken the water surface tension to allow the water to flow. The impact of PSMs in reducing the water surface tension and colony spreading were also verified using the *psmα* deletion mutant CGL007 (Figs 2A and 4Bc). These phenomena are strikingly similar to colonies formed by a swarming bacterium, *B. subtilis* F29-3. When this bacterium is cultured on LB agar containing 0.7% agar, the bacterium extracts water from the medium and uses surfactin to cause water to flood agar surface to facilitate swarming^16^. This study suggests that *S. aureus* HG001 uses a similar mechanism to spread on TSA.

Since *S. aureus* lacks a flagellum and an apparatus that would allow the bacteria to move actively as the swarming bacteria do, *S. aureus* likely moves passively on TSA medium. If bacteria move actively, they should move in different directions with different speeds in a fluid filled environment. However, in a colony that was formed on TSA-0.4 medium, all the *S. aureus* HG001 cells were moving in one general direction with the same speed (Supplementary Video S2, S3). These results suggest that *S. aureus*, rather than moving actively, was carried by flowing water across the water surface. Meanwhile, we found that *S. aureus* was attached to the surface of TSA-0.4 medium. *S. aureus* gradually floated in a colony after being cultured for more than 1 h (Fig. 5). This finding also suggested the bacteria lack active movement, resulting in floating and drifting in water.

The force that creates water flow likely comes from a continuous water extraction by the bacteria and a weakening of the water surface tension of a spreading colony by PSMs. The transcription of PSM genes is activated by quorum sensing^9^,^19^ and is required for spreading^5^,^6^,^7^, suggesting that quorum sensing occurs in a spreading colony. Our confocal laser-scanning microscopic work showed that GFP is not expressed much by the colony of HG001(pPSM-gfp) at Hours 1 and 2 but is strongly expressed after Hour 3 (Fig. 6A). Immunoblot analysis also verified that the expression of GFP started to increase at Hour 3 (Fig. 6B), suggesting that quorum sensing and transcription of the PSM genes start before or at Hour 3. It is likely that cells in the entire colony are transcribing *psm*α as an earlier study showed that *agr*P3 activity was similar among the cells between the center and edge in a spreading colony^21^, thus the expression of PSMs in different areas of the spreading colony may be similar. The edge of the colony showing stronger green signal (Fig. 5) may be due to the accumulation of cells by the coffee ring effect^22^,^23^.

Based on the results from this study, we propose a mechanism to explain how *S. aureus* spreads. This mechanism involves a continuous water extraction from the medium. As the bacterial density of the colony increases, quorum sensing occurs, which triggers the transcription of *psm* genes and the synthesis of PSMs. These biosurfactants weaken the water surface tension of the colony allowing water to flow. The water flow then carries the bacteria and causes a rapid spreading of the bacteria across the agarose surface. In nature, an ability to move rapidly gives bacteria advantages to obtain food^24^ and is an important virulence factor for many pathogens^25^,^26^,^27^,^28^. An earlier study showed that *S. aureus* is capable of spreading on fresh pork meat^6^, showing that this organism is able to spread on soft tissues. Our study reveals how *S. aureus* spreads, which is important in the understanding of how this pathogen moves in human tissues to cause diseases.

## Materials and Methods

### Strains and culture conditions

*S. aureus* HG001^29^, a derivative of *S. aureus* NCTC8325, is able to spread on soft agar. *S. aureus* CGL005 is a mutant strain derived from strain HG001, which has a transposon inserted in *agrD* (Supplementary Fig. S1). *S. aureus* CGL007 is a mutant strain with a deletion in *psmα* operon. Tryptic soy broth (TSB) and tryptic soy agar (TSA) were used to culture *S. aureus*. TSA plates were prepared 20 min before inoculation according to the method of Kaito and Sekimizu^1^, except that the plates were prepared with agarose from Seakem (Lonza Rockland, Inc.). Bacteria were cultured in TSB overnight, and washed in PBS. The plates were inoculated with 2 μl (1 × 10^7^ CFU) of the bacteria and then cultured at 37 °C. Strains containing pRPO-gfp or pPSM-gfp were cultured on TSA plates supplemented with 10 μg/ml chloramphenicol. PSMα3 was synthesized chemically by Kelowna International Scientific Inc. (Taiwan) and was applied (2 μl, 1 mM) to the center of 25 ml TSA-0.4 plates. After drying for 30 min in a hood, the plate was inoculated with CGL005 or CGL007 at the same location to demonstrate that PSMα3 is required for spreading by the mutant strains.

### Transposon mutagenesis

To identify the genes that are involved in colony spreading, *S. aureus* HG001 was mutagenized with a mariner-based transposon, *bursa aurealis*, according to a method described elsewhere^17^. The transposon contains the short inverted repeats of the hornfly transposon and the *ermB* resistance marker. Insertion of *bursa aurealis* into the genome resulted in resistance to erythromycin. *S. aureus* HG001 were sequentially transformed with pBursa and pFA545, which contains genes encoding mariner transposase and confers resistance to tetracycline and ampicillin. The resulting transformants were spread on TSA containing erythromycin (TSAerm) and incubated at 30 °C for 24 hr. After induction of transposase expression and curing the plasmid by incubating the transformants at 42 °C for 2 days, cells containing transposon insertion in the chromosome were selected on TSAerm. Approximately 2000 mutants were collected. Mutants that were defective in spreading were selected on TSA medium that contained 0.25% agarose (TSA-0.25). The site of transposon insertion was determined by inverse PCR using primers that were complementary to the border sequences of the transposon according to a method described elsewhere^17^. The amplified DNA fragment was then sequenced to identify the location of transposon insertion, using the genome sequence of *S. aureus* NCTC8325 (accession number: NC_007795.1)^30^, which is the parental strain of *S. aureus* HG001.

### Construction of PSM mutant strain of *S. aureus*

To generate *psmα* deletion mutant, gene replacement using the temperature-sensitive plasmid pMAD was performed according to the method described elsewhere^31^. A 1.3-kb DNA fragment containing the entire spectinomycin-resistance gene *spc*^31^ was amplified using the primer Spc-F (5′-CCGCTCGAGAGTAGTTCACCACCTTTTC) and Spc-R (5′-CGCCCCGGGTTTATTGTTTTCTAAAATC). The PCR product was digested with XhoI and SmaI and inserted into the same sites in pGEM-7Z to generate p7Z-spc. The upstream and downstream regions of *psmα* operon were amplified by PCR using the primer pairs Up-F1 and Up-R1 (5′-GATGAGCTCATAATGTAATACCCCAGCAG and 5′-CTACCCGGGATAGTTATCTTGTGCGTAAT), Dn-F1 and Dn-R1 (5′ GAACTCGAGTTCTCAGGCCACTATACCAA and 5′ GCGGCATGCACAATACAATCACGTAGCAT), respectively. The PCR products were cut using SacI, SmaI, XhoI and SphI, and inserted into the SacI-SmaI and XhoI-SphI sites in p7Z-spc to generate p7Z-007. The fragment containing *spc* flanked with the upstream and downstream regions of *psmα* operon was amplified from p7Z-007 using primer Up-F2 (5′-GATGTCGACATAATGTAATACCCCAGCAG) and Dn-R2 (5′-GCGAGATCTACAATACAATCACGTAGCAT). The PCR product was digested with SalI and BglII and inserted into the same sites in pMAD to generate pMAD-Δpsmα. Allelic replacement of the *psmα* operon by a spectinomycin cassette (*spc*) was performed by introducing the pMAD-Δpsmα into *S. aureus* strain HG001 to generate the *psmα* mutant strain CGL007 according to the method described previously^31^.

### Plasmid construction

The promoter of *rpoD* gene of *B. subtilis* was amplified by PCR, using primers 5′-CGCGTCTAGAATCAACAGAATCAAAGAGGA and 5′-CGCGGGATCCATGTATATGAATTTGTCGAA. The amplified fragment was cut with BamHI and XbaI and inserted at the BamHI-XbaI sites in pRU-gfp^16^ to generate pRU-RPOD. A DNA fragment that contained the *rpoD* promoter along with the *gfp* sequence in pRU-RPOD was isolated by EcoRI and XbaI digestions, and the fragment was inserted into the same sites in a shuttle vector pGHL6^32^ to generate pRPO-gfp. A DNA fragment that contained the *psm*α promoter was amplified using primers 5′-GGTCTAGAATGAGCTTAACCTCTATTAA and 5′-CAAGGATCCGCTTATGAGTTAACTTCAT, and *S. aureus* HG001 DNA was used as a template. The DNA fragment was cut by BamHI and XbaI and then inserted into the BamHI-XbaI sites in pRPO-gfp to generate pPSM-gfp. To complement the mutants CGL005 and CGL007, pGH-agr and pGH-psm were constructed. The vector pGHL6 was cut with EcoRI and self-ligated to generate pGHL7, which was used to construct pGH-agr and pGH-psm. A fragment containing *agrB*, *agrD*, *agrC* and their promoter was amplified by PCR, using primers Agr-F (5′-AGCTTCTAGACTGAGTCCAAGGAAACTAAC) and Agr-R (5′-AGCTGAATTCGGATCGTCTTCGCAAATGAA) and *S. aureus* HG001 chromosome as a template. The PCR product was then cut with XbaI and EcoRI, and inserted into the XbaI-EcoRI sites in a vector, pGHL7 to generate pGH-agr. The *psmα* operon with its promoter was amplified by PCR, using primers PSM-F (5′-GGTCTAGAATGAGCTTAACCTCTATTAA) and PSM-R (5′-GGAGATCTTTAAGTATTCAATTCAATTCGCTTA) and the *S. aureus* HG001 chromosome as a template. The PCR product was cut with XbaI and BglII and inserted into the XbaI-BglII sites in pGHL7 to generate pGH-psm.

### Microscopy

Bacterial colonies were observed under a Zeiss Axioskop phase contrast microscope at a magnification of 400x. Bacterial movement was recorded using a Sony NEX-5N camera. To measure the height of colonies, *S. aureus* HG001(pRPO-gfp), *S. aureus* CGL005(pRPO-gfp) and *S. aureus* CGL007(pRPO-gfp) were inoculated on 25 ml TSA-0.4 plates that contained 1/100th of its volume of 0.5 μm carboxylate-modified FluoSphere beads (Invitrogen, Ex580/Em605) and cultured at 37 °C for indicated time. At each time point, one plate was moved out from the incubator, cover removed, and placed on the stage of an upright microscope for observation. Different plates were used for the indicated time point. A Z stack in which the planes were separated by 10 μm was acquired using an upright laser-scanning confocal microscope (Leica, TCS-SP2). Green fluorescence indicated the distribution of bacteria in the spreading colony. The last plane showing red fluorescence was used to indicate the plate surface. The height of the colony was determined by green fluorescence using the method described elsewhere^16^. 3-D images of colonies were generated from the Z stacks using Imaris software.

### Gravity experiments

To demonstrate that colonies contained water, plates were tilted at 30° during incubation to allow water and bacteria to flow out of the colonies according to the method described earlier^16^.

### Quorum sensing

TSA-0.4 plates were inoculated with 2 μl PBS-washed log-phase bacteria (approximately 1 × 10^7^ CFU). Green fluorescence exhibited by the colony was observed under a confocal laser-scanning microscope. To determine the amount of GFP expression, 100 μl of the bacteria was applied to TSA plates. The bacteria and medium were removed using a surgical blade, vortexed, and harvested by centrifugation^16^. The expression of GFP was examined by immunoblotting using the method described earlier^16^.

## Additional Information

**How to cite this article**: Lin, M.-H. *et al.* Modulation of *Staphylococcus aureus* spreading by water. *Sci. Rep.* **6**, 25233; doi: 10.1038/srep25233 (2016).

## Supplementary Material

Supplementary Video S1

Supplementary Video S2

Supplementary Video S3

Supplementary Information
