# Supplementary material for: Modulation of Staphylococcus aureus spreading by water
Source: Sci Rep. 2016 Apr 29;6:25233. doi: 10.1038/srep25233 (PMC4850448; doi:10.1038/srep25233)
Supplement: Supplementary Information [file srep25233-s4.pdf]

Supplementary information

Modulation of *Staphylococcus aureus* spreading by water

Mei-Hui Lin[1,2,3,#]*, Wan-Ju Ke[3,4#], Chao-Chin Liu[1] and Meng-Wei Yang[1]

[1]Department of Medical Biotechnology and Laboratory Science, College of Medicine, Chang-Gung University, Taoyuan, 333, Taiwan

[2]Department of Laboratory Medicine, Chang-Gung Memorial Hospital, No. 5, Fusing St., Guishan, Taoyuan, 333, Taiwan

[3]Research Center for Bacterial Pathogenesis, Chang-Gung University, Taoyuan, 333, Taiwan

[4]Department of Microbiology and Immunology, Chang-Gung University, Taoyuan, 333, Taiwan

Wan-Ju Ke: ruru650316@gmail.com

Chao-Chin Liu: k1e3y5@hotmail.com.tw

Meng-Wei Yang: sakura8119@kimo.com

*Corresponding author: Mei-Hui Lin, E-mail address: thea@mail.cgu.edu.tw

TEL: +886-3-2118800 ext: 5206

FAX:+886-3-2118292

[#]These authors contributed equally to this work.

Supplementary Figure S1.

```
ATGAATACAT TATTTAACTT ATTTTTTGAT TTTATTACTG GGATTTTAAA   50
 M  N  T   L  F  N  L   F  F  D   F  I  T   G  I  L  K

                           ↓
AAACATTGGT AACATCGCAG CTTATAGTAC TTGTGACTTC ATAATGGATG   100
  N  I  G   N  I  A   A  Y  S  T   C  D  F   I  M  D

AAGTTGAAGT ACCAAAAGAA TTAACACAAT TACACGAATA A            141
E  V  E  V   P  K  E   L  T  Q   L  H  E  •
```

Figure S1. The sequence of *agrD*. Arrow indicates the site of *bursa aurealis* transposon insertion in CGL005. The amino acid sequence is shown under the DNA sequence. The number indicates nucleotide position, starting from the initiation codon of *agrD*. The dot represents termination codon.

Supplementary Video legends

Supplementary Video S1. Brownian movement of *S. aureus* HG001 at 20 min after inoculation. Two microliters of the bacteria was inoculated on 25-ml TSA-0.4 plates. Movement of the bacteria was observed under a phase contrast microscope at a magnification of 400x. This movie shows that bacteria were distributed sparsely within the colony. Many bacteria were found oscillating, typical of Brownian movement.

Supplementary Video S2. Movement of *S. aureus* HG001 at 2.5 h after inoculation. This movie shows that a few bacterial cells stick on the plate surface; most bacteria were floating and drifting in flowing water toward a general direction.

Supplementary Video S3. Movement of *S. aureus* HG001 at 4 h after inoculation. The movie shows that bacteria in the colony were densely packed and flowing in the same directions.
